# Analysis of Political Documents and Recommendations in Terms of Physical Activity Promotion for People with (Intellectual) Disabilities

**DOI:** 10.3390/healthcare13222838

**Published:** 2025-11-08

**Authors:** Simone Neumeister, Christoph Kreinbucher-Bekerle

**Affiliations:** Institute of Human Movement Science, Sport and Health, University of Graz, 8010 Graz, Austria

**Keywords:** document analysis, exercise, sport, health-enhancing physical activity, guidelines, easy-to-read language

## Abstract

**Background/Objectives:** Compared to the general population, people with (intellectual) disabilities often exhibit a greater degree of sedentary behavior and inactivity. Therefore we systematically examined various political and institutional guidelines, directives and frameworks for the promotion of physical activity (PA). The aim of this empirical study was to conduct a document analysis of various documents using previously defined criteria to examine the occurrence of terms and objectives relating to the promotion of PA. **Methods:** The sample (N = 19) included 13 Austrian and 6 international policy and strategy concepts for promoting PA as well as legal frameworks and service specifications for facilities and support services for people with (intellectual) disabilities. To obtain a systematic overview of the frequencies of terms and objectives of PA promotion in selected documents, a frequency analysis procedure was used. Moreover, all documents were screened based on availability in easy-to-read language, which is essential for people with intellectual disabilities. **Results:** The results of the analysis show a varied distribution of the frequency of references to PA promotion within the sample. International guidelines and World Health Organization (WHO) documents, as well as documents on national strategies for promoting PA behavior in Austria, contain relevant terminology and objectives for PA promotion. **Conclusions:** However, the promotion of PA is barely mentioned in some of the analyzed documents, as are related terminology and goals. These should be placed more at the center of political and social measures to raise awareness of health-enhancing PA for people with and without (intellectual) disabilities.

## 1. Introduction

Despite extensive evidence on the health benefits of regular physical activity (PA) for all people [1], only 9% of people with intellectual disabilities meet public PA recommendations [2]. This may be due to limited access to accessible formats or a lack of knowledge among caregivers [3]. Various studies on PA behavior and the general health situation of people with intellectual disabilities show that promoting health-enhancing PA is particularly relevant for this target group [4,5]. People with intellectual disabilities can have limitations in intellectual and cognitive performance, as well as in social and adaptive behavior [6]. Moreover, the comorbidity with other types of disabilities is high, e.g., physical or psychological [7]. This can lead to a reduced ability to cope with everyday tasks independently. Promoting cognitive functions and strengthening practical everyday and social skills are just two of the many benefits of regular PA [8,9].

People with intellectual disabilities are more frequently overweight and obese than the general population [4]. Furthermore, this target group has an increased risk of various diseases such as cardiovascular disease, cancer, and type 2 diabetes [4,10]. Regular PA promotes health and reduces the risk of these diseases. An active lifestyle also has a positive effect on cognitive functions and psychosocial well-being [8,9]. Regular PA can not only reduce the risk for depression and anxiety, but also counteract social isolation, for example, through participation in community sports activities [11,12]. However, there are limited offers for people with intellectual disabilities except within the Special Olympics movement. Thus, people with intellectual disabilities generally exhibit lower overall health indicators and spend more time being sedentary [13].

Therefore, PA promotion is a key tool for comprehensively improving the health-related quality of life of people with and without intellectual disabilities. Participation in PA and sports is a human right and anchored in the article 30 of the UN Convention on the Rights of Persons with Disabilities (UNCRPD), which represents a crucial aspect of the inclusion of people with disabilities in general [14]. According to the World Health Organization (WHO) recommendations [15], adults with and without disabilities should engage in at least 150–300 min of moderate-intensity, or 75–150 min of vigorous-intensity, aerobic PA as well as muscle-strengthening activities two times per week [16]. Physical activity guidelines play a central role for the promotion of health and lead to policy related recommendations [17]. National action and strategy concepts, often based on international guidelines, are also an important influence in promoting PA awareness and the development of sustainable behaviors [18]. They provide essential information on PA recommendations and goals and serve as a basis for the implementation of PA promotion at political and societal levels. However, there is a lack of evidence and documents are mostly not accessible or written in easy-to-read language, which would be mandatory for people with intellectual disabilities, helping to break down communication barriers.

Despite the existence of PA recommendations [15] and the recognition of participating in PA and sport as a basic human right [14], these measures often fail to reach people with intellectual disabilities. This can be attributed to existing reservations about people with disabilities. In a study [5] investigating the relationship between caregivers’ PA behavior and their assessment of the PA needs of people with intellectual disabilities, 47.1% of caregivers suggested less than the recommended 150 min of PA per week [15] for people with intellectual disabilities, although being sufficiently active themselves.

Policy documents and guidelines are of central importance because they play a key role in designing and implementing social, educational and health measures and concepts [19]. However, the knowledge of the general population as well as caregivers of people with intellectual disabilities on public health recommendations on PA is limited [3]. To date, to the best of our knowledge, there is no study reflecting on PA promotion in political documents or guidelines related to the target group of people with (intellectual) disabilities. Therefore, our aim was to investigate the extent to which the terms and objectives of the promotion of PA are embedded in selected country-specific and international documents (N = 19), with the country Austria as a prime example. To provide an overview of the extent to which terminology and objectives for promoting PA are anchored in selected international and national handbooks and guidelines, a document analysis was conducted. The article therefore refers to people with (intellectual) disabilities, as measures and strategies to promote PA for people with disabilities also benefit the sub-group of people with intellectual disabilities. The analysis was also expanded to examine whether the documents in the sample are available in easy-to-read language in order to assess their accessibility for the target group of people with intellectual disabilities.

## 2. Materials and Methods

### 2.1. Research Design

The main objective of this study was to determine the extent to which the promotion of PA for people with (intellectual) disabilities is anchored in specific documents.

The document analysis reveals varying frequencies in the mention of terms and objectives and identifies selected documents in which action is needed to further integrate PA promotion. Particularly regarding the target group of people with intellectual disabilities, it was also investigated whether the documents are available in plain or easy-to-read language and whether there is easy online access.

As part of the document analysis and the conception of a systematic overview analysis of the terminology and objectives of PA promotion used in selected documents, specific criteria for document selection were established at the outset. The establishment of these criteria served as a basis for the analysis and was particularly relevant given the initial situation, in which certain international documents—such as the UN Convention on the Rights of Persons with Disabilities [14] and the WHO guidelines [15,20]—were of particular interest to the research project.

Based on this, additional criteria for sample selection were derived, which are explained in more detail in the following description of the sample (see Section 2.2). National documents from Austria were also included in the sample to facilitate the comparison between international documents and Austrian handouts and guidelines.

### 2.2. Sample Description

The analytical approach involved using standard search engines, such as Google and Google Scholar, to identify the documents to be included in the sample. Search terms such as “promotion of physical activity (in Austria)” or “promotion of physical activity for people with disabilities” were used to search for documents on the Internet. All the documents in the sample were also identified by referencing to other relevant publications, which involved searching the documents and bibliographies. The WHO document “Global Strategy on Diet, Physical Activity and Health” from 2004 [21] was one of the first documents included in the sample. For the further description of the sample, the year 2000 was therefore chosen as the starting point in order to narrow down the selection of the sample accordingly. In addition to being published after the year 2000, further criteria for the sample description included that the documents had to be official PDF files that are freely accessible (open access) and available in German or English language. Thus, a total of 19 documents were identified that are of relevance both to promoting PA among the general population and to the personal, institutional, and political lives of people with disabilities at international (n = 6) and national (n = 13) levels.

### 2.3. Data Analysis

The analysis was carried out between February and March 2025. A frequency analysis according to Mayring [22] was conducted, recording the frequency of coded segments. A category system was developed in advance for the systematic coding of the data. To analyze the documents specifically in terms of the terminology and objectives of PA promotion, Impact Goal 1 from Health Goal 8 [23] document was used as the basis for forming the main categories. This is because the concept of promoting PA and the associated objectives are comprehensive, and the main categories were defined in this study based on this relevant literature [23]. This impact objective from Health Goal 8 [23] describes the importance of knowledge about the positive health effects of PA, the health-promoting levels of PA, and the various possibilities and offerings of PA for effective PA promotion [23]. Based on this the following main categories were formed: *Promoting PA, Knowledge, Forms and types of PA*, and *PA settings.*

Various subcategories were also formulated using a systematic approach. For example, the subcategories of the main category *Knowledge* were based on the Austrian PA recommendations [24], which in turn are based on the WHO guidelines [15]. These guidelines provide recommendations for different target groups. If coded segments could not be assigned to a target group-specific subcategory, they were assigned to the subcategory *General/non-group-specific knowledge*. Compared to the other main categories, larger text sections were coded here, with a coded paragraph comprising a maximum of one paragraph and capable of containing multiple sentences, provided they contained information on the positive effects of regular PA. If the information extended beyond one paragraph, a new code was performed.

**Figure 1:**
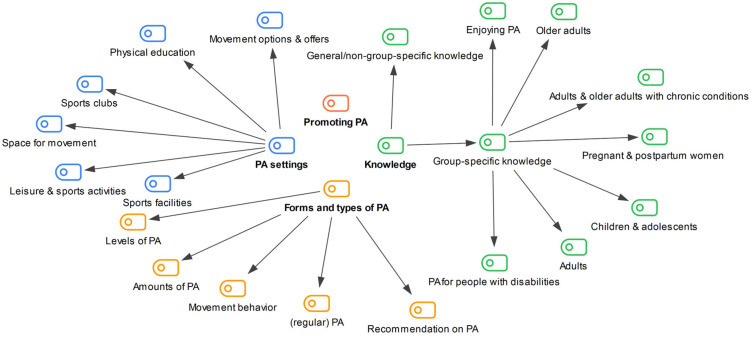
Category system of the document analysis with the four main categories *Promoting PA (depicted in red), Knowledge (green), Forms and types of PA (orange)* and *PA settings (blue)*. Note: The different colors demonstrate presence in the relevant main categories.

A systematic approach was also taken for the subcategory *PA settings*. Based on the outcome of objective 1 of Health Goal 8 [23] and the question of where PA takes place, terminology was derived from Article 30, paragraph 5 of the UNCRPD [14], which enshrines the participation of people with disabilities in recreational, leisure, and sporting activities. The category system for the document analysis was developed on this basis and can be found in Figure 1.

The data collection and coding using the category system was carried out using MAXQDA (version 24.7.0) in two steps. First, the documents were searched for specific subcategory terms using the MAXQDA search function, after which initial coding was carried out. The documents were then read in full to identify any additional relevant segments that had not been identified by automatic search. This procedure was chosen to ensure that all relevant codes were recorded as thoroughly as possible. Since qualitative content analysis [22] was used, in addition to a category system, a correspondence table was created with examples to illustrate the segments to be coded within a category (see Table 1). This was particularly relevant due to the inclusion of documents in English and German language and the different coding units.

As easy-to-read language is essential for people with intellectual disabilities, the documents in the sample were also examined for their availability in easy-to-read language (see Section 3.5). For the internet search, the title of the publication was first entered into common search engines, such as Google, to check if an easy-to-read version was available. If no relevant publications were found, the search was expanded to include the term ‘easy-to-read language’ to see if there were any alternative publications with similar content written in plain language. Documents were classified as ‘YES’ if they were available in easy-to-read language and were clear and easy to find. They were classified as ‘Potentially’ if accessibility for the target group could be improved by providing an easy-to-read version or by making the document easier to find. A classification of ‘NO’ was given if the document was not available in easy-to-read language or was unclear or difficult to find. For the category ‘Potentially’, justifications (categories A, B, and C) were developed to clarify and facilitate understanding of this classification.

## 3. Results

### 3.1. Overall Results of the Frequency Analysis

A total of N = 19 documents were analyzed regarding the terminology and objectives of PA promotion. In these documents, 2636 segments were coded. There were no coded segments in four documents, meaning that these documents do not make any reference to promotion of PA. These included two Austrian documents on legal guidelines and framework conditions for people with disabilities in Austria [26,27] as well as one document on Austria’s health goals, which aims to strengthen the health literacy of the population [28]. Another document, which was not assigned a code, is a document on the interdisciplinary topic of “disability” in Austria [29]. Therefore, these four documents were not included in the further presentation. Thus, 15 of 19 (78.95%) documents made references to the promotion of PA; however, the references differ in quantity and quality.

To obtain a better overview of the distribution of the results in the different documents, four document groups were formed from the sample, which contain the following number of documents with references to promoting PA: “WHO guidelines and documents” (5), “Legal frameworks and service descriptions” (3), “Austrian documents on PA and disabilities” (5) and “Austrian health goals” (2).

### 3.2. Distribution of the Document Groups

**Figure 2:**
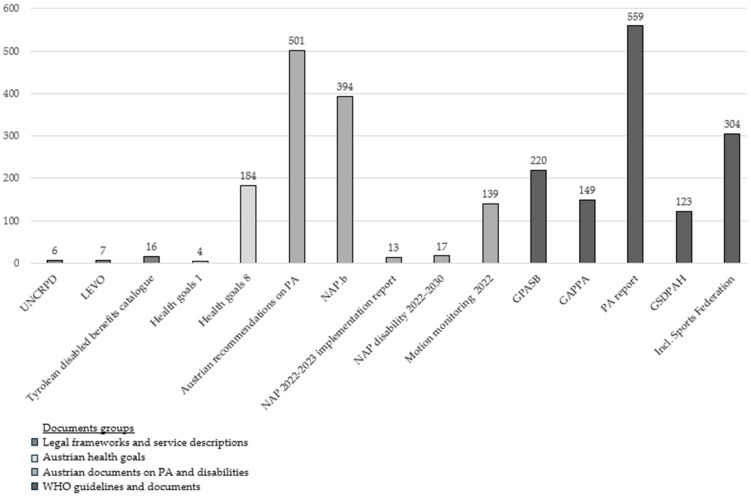
Distribution of documents and frequencies of coded segments; n = 15 documents Note: The four documents in which no references for promoting PA could be identified were not included in the figure. Table 2 lists the citations of the analyzed documents. The abbreviations used in the article are listed on page 15.

As can be seen in Figure 2, the documents in the “Legal Framework and Service Descriptions” document group, with a total of 29 coded segments, exhibit the lowest frequency according to the predefined category system for analyzing terminology and objectives of PA promotion. This corresponds to a percentage share of 1.10%. In contrast, terminology and objectives related to PA promotion appear in the “Austrian Health Goals” document groups at 7.13%, “Austrian Documents on PA and disabilities” at 40.36%, and “WHO Guidelines and Documents” at 51.40%. The four documents in which no references for promoting PA could be identified were not included in the following figure.

### 3.3. Overview of the Results of the Frequencies of the Coded Segments

Table 2 above shows the documents in the sample (n = 15) along with the frequencies of the coded segments.

The documents with the most coded segments can be assigned to the document groups “Austrian documents on PA and disabilities” and “WHO guidelines and documents.” “The Global Status Report on Physical Activity” [20] was coded most frequently with 559 references, followed by the Austrian PA Recommendations [24] with 501 and the National Action Plan for PA (NAP.b) [33] with 394, which is a National Action Plan on PA in Austria.

Other documents that frequently refer to terminology and objectives for PA promotion in their publications are the documents “Inclusive, sustainable, welcoming national sports federation” [38] with 304 coded segments and the “Guidelines on Physical Activity and Sedentary Behaviour” [15] with 220 coded segments. One reason why the Austrian PA recommendations [24] and the “Guidelines on Physical Activity and Sedentary Behaviour” [15] have a difference of 281 coded segments, although the former is based on the WHO guidelines on PA recommendations, lies primarily in the categories of *Knowledge* and *Forms and types of PA*. For example, the WHO’s PA recommendations [15] for people with disabilities are described in a separate chapter, while in the Austrian PA recommendations [24], the target group in the recommendations encompasses several cohorts by referring to people with and without physical, sensory, or intellectual disabilities. Due to this characteristic, the knowledge category was coded for the respective age group of the recommendations and additionally for the subcategory of people with disabilities.

### 3.4. Results by Main Categories

As already shown in Figure 2, four main categories were created: *PA promotion*, *Knowledge*, *Forms and types of PA*, and *PA settings*. The main category of *Forms and types of PA* was coded most frequently, with a total of 1560 segments (59.18%). The comparatively large gap between the other main categories—*PA settings* (550, 20.86%), *Knowledge* (258, 9.79%), and *PA promotion* (262, 9.94%)—is explained by the inclusion of the subcategory of *(regular) PA* in the category system. Since the sample included numerous documents on PA-promoting measures and strategies, as well as recommendations for health-promoting PA, many coded segments could be assigned to this subcategory.

#### 3.4.1. PA Promotion

In total, 13 of 15 documents included in the frequency analysis had at least one segment assigned to the main category of *PA promotion*. The main category of *PA promotion* was coded most frequently in the document NAP.b [33], with a total of 113 references (see examples in Table 3).

#### 3.4.2. Knowledge

The main category of *Knowledge* was coded in eleven documents. According to the criteria of this analysis, eight documents contained no references to general or target group-specific knowledge on the physical or psychosocial health benefits of PA. The documents were specifically searched for knowledge on the health-promoting effects of PA. References for health promotion were only coded if there was a clear connection to PA. Segments related to workplace health promotion were not included.

Five documents each contained references to knowledge on people with disabilities and on the effects of PA on cognitive functions. These two subcategories are presented in more detail below due to their relevance for people with intellectual disabilities.

##### Subcategory: PA for People with Disabilities

A total of 36 references to target group-specific knowledge on the health-promoting factors of regular PA were found in five documents in the sample. The frequencies of the coded segments in the subcategory *People with Disabilities* are distributed across the five corresponding documents as follows: “Austrian recommendation on PA” [24] (18), “Guidelines in Physical Activity and Sedentary Behavior” [15] (9), “NAP.b” [33] (7), “National Action Plan on disability 2022–2030” [35] (1), and “Global Status, Report on Physical Activity” [20] (1). The document on the Austrian recommendations on PA contains significantly more references than the other documents mentioned, as it includes people with disabilities in almost all target group-specific recommendations and therefore double coding was carried out. Table 4 below shows examples of coded segments in the PA subcategory *PA for people with disabilities*.

#### 3.4.3. Forms and Types of PA

The main category of *Forms and types of PA* was the most frequently recorded, with 1560 codes, accounting for 59.18% of all references. In contrast to the main category *Physical activity promotion*, which searches for specific terms such as ‘physical activity promotion’ or ‘promoting physical activity’, the main category *forms and types of PA* comprises defined subcategories that cover specific terms such as ‘physical activity behaviour’ and ‘physical activity recommendations’. These subcategories provide information on how PA should be organized and how frequently it should be undertaken.

The most codes were found in the “Global Status Report on Physical Activity” [20] with 493 codes and the document on Austrian recommendation on PA [24] with 332 codes. The subcategory of *(regular) PA* was the most frequently coded in the main category *Forms and types of PA* with 43.21%, significantly more than the subcategories *Amount of PA* (0.80%), *Recommendation on PA* (9.03%), *Levels of PA* (4.70%), and *Movement behavior* (1.44%). Of the 15 documents with references to PA promotion, only one document contained no references to the main category *Forms and types of PA.* The subcategory of *regular PA* was the most frequently recorded, with 1139 codes. This is because many documents in the sample contain measures and strategy concepts to promote PA awareness, which is why terms such as “physical activity”, “regular physical activity” and “physical exercise” occur more frequently depending on the context.

#### 3.4.4. PA Settings

Of the 15 documents examined, 13 contained references to the main category of *PA settings.* The highest number of codes within this main category, namely 262 references, was made in the WHO guideline “Inclusive, Sustainable, Welcoming National Sports Federations” [38], which is due to the thematic focus of the guideline.

More than half (58.7%) of the coded segments in the main category of *PA settings* fall into the subcategory of *sports clubs*. The WHO guideline “Inclusive, Sustainable, Welcoming National Sports Federations” [38] contains 252 codes, Health Goal 8 [23] contains 25, and NAP.b [33] contains 29 codes.

### 3.5. Examination of the Documents for Easy-to-Read Language

Easy-to-read language is an important resource for promoting PA, as it makes knowledge and information accessible to people with intellectual disabilities and enables them to inform themselves about PA-related topics. For example, recommendations and advice on the health-promoting effects of PA can be better understood. The understandable presentation of knowledge is becoming increasingly important in this area.

For these reasons, the documents in the sample were also examined for their availability in easy-to-read language. However, it became apparent that relevant publications were often not directly accessible in easy-to-read language or could only be found through more intensive research, by adding the search term ‘easy-to-read language’ and therefore classified as ‘Potentially’. Justifications (categories A, B, C) were also formulated to make the classification as ‘Potentially’ easier to comprehend.

For example, documents Health Goal 1 [32] and Health Goal 8 [23] are classified as ‘Potentially’ (category A) because, instead of separate documents, concise summaries of Austria’s health goals are provided with a reduced scope of content. Similarly, the document on Austrian PA recommendations provides important guidelines for improving individuals’ PA behavior. However, as can be seen from the following Table 5, the official document containing the Austrian PA recommendations [24] is marked as ‘Potentially’ (category B), because an extended internet search using the term ‘easy-to-read language’ yielded two publications [39,40] that presented the central content of the recommendations in an accessible way. In addition, the NAP 2012–2020 is classified as ‘Potentially’ (category C) because there is no easy-to-read version of the NAP 2022–2030 [35]. However, the Federal Minister for Labor, Social Affairs, Health, Care, and Consumer Protection of the Republic of Austria is currently preparing an easy-to-read version of the NAP 2022–2030 [41].

Efforts to provide health-related information in easy-to-read language are valuable and appreciated, as they enable people with lower literacy skills to access important content. From the authors’ perspective, a few targeted adjustments could significantly increase the accessibility of documents marked as ‘Potentially’, thereby enabling their valuable content to reach a broader target group, including people with intellectual disabilities.

In this context, particular mention should be made of the German translation of the UN Convention on the Rights of Persons with Disabilities [30]. It is the only document marked ‘Yes’ on the table below. This means that, when checking whether the document was also available in an easy-to-read version, entering the title in the search field was sufficient. On the website of the Austrian Federal Minister for Labour, Social Affairs, Health, Care and Consumer Protection [42], a link to the easy-to-read version was found directly below the original German translated UNCRPD document. Therefore, an additional search using the term ‘easy-to-read’ was not necessary in this case.

The following Table 5 provides an overview of the documents and the results of this review.

**Table 5:** Results of the examination of the documents for easy-to-read language.

Document Type *	Document	Easy-to-Read Language ** and Justification ***
1	UNCRPD (2016) [30]	YES
1	LEVO (2015, version 2025) [25]	NO
1	Tyrolean disabled benefits catalogue (2015) [31]	NO
2	Health goals 1 (2017) [32]	Potentially—category A
2	Health goals 8 (2022) [23]	Potentially—category A
3	Austrian recommendations on PA (2022) [24]	Potentially—category B
3	NAP.b (2024) [33]	NO
3	NAP 2022–2023 implementation report (2024) [34]	NO
3	NAP disability 2022–2030 (2022) [35]	Potentially—category C
3	Motion monitoring 2022 (2022) [36]	NO
4	GPASB (2020) [15]	NO
4	GAPPA (2018) [37]	NO
4	PA report (2022) [20]	NO
4	GSDPAH (2004) [21]	NO
4	Incl. Sports Federation (2023) [38]	NO

Note: * Document type: 1 = Legal frameworks and service descriptions, 2 = Austrian health goals, 3 = Austrian documents on PA and disabilities, 4 = WHO guidelines and documents ** YES = The document is also available in a version in easy-to-read language and is clear/easy to find. Potentially = Improving accessibility for the target group could potentially be beneficial. NO = The document is not available in easy-to-read language or is not clear/easy to find. *** Additional explanations as to why something is classified as ‘Potentially’: category A = Instead of a separate document, a concise summary of Austria’s health goals is provided, with a reduced scope of content; category B = There is no separate document that exclusively presents PA recommendations in an easy-to-read format. However, there are other publications that contain content on PA recommendations in easy-to-read language, as well as covering other health-promoting aspects (e.g., [40]); category C = While a version of the National Action Plan 2012–2020 is available in easy-to-read language, there is no corresponding edition of the NAP 2022–2030 [35]. However, the Federal Minister for Labor, Social Affairs, Health, Care, and Consumer Protection of the Republic of Austria is currently preparing an easy-to-read version of the NAP 2022–2030 [41].

## 4. Discussion

The current paper reflects on the mentioning of PA in current policy documents and guidelines. Therefore, a document analysis was conducted with 19 documents, both international as well as country-specific documents for the situation in Austria. The promotion of PA has positive health-related effects, including a reduced risk of various diseases such as cardiovascular disease, type 2 diabetes, obesity, and cancer [1]. This is particularly important for people with intellectual disabilities, who are at higher risk than the general population [4,10]. Regular PA also demonstrably contributes to promoting general health and improving quality of life [9]. Despite the positive effects of PA, the documents examined in this investigation show varying frequencies of PA-related terminology. Four of the 19 documents did not mention aspects of PA at all, although some of these documents were related to health and well-being of people with disabilities.

A key international document included in the analysis is the UNCRPD [14]. It is of great importance for the equal participation of people with disabilities in all areas of life—including PA and sport. The German translation was used for the analysis. However, the study revealed that only six segments of the document could be assigned terminology and objectives related to promoting PA. This is in line with the work of Aichele [43], who also points out the limited substantive development of the topic of sport and PA in the UNCRPD, but emphasizes its central importance for equal opportunities for people with disabilities in the field of sport and PA promotion.

Other documents with low frequencies in the analysis come from the same document group “Legal framework and service descriptions.” This includes both the legal basis for equal rights and participation of people with disabilities in Austria and two country-specific service descriptions for disability assistance. Overall, these documents contain only 29 references to PA promotion, significantly fewer than the groups “Austrian documents on PA and disabilities” with 1064 and “WHO guidelines and documents” with 1355 coded segments on corresponding objectives and terminology.

The analysis shows that Austrian performance descriptions in care services for people with disabilities are not fully comparable, as they are defined at a federal-state level. Similar comparative analyses with other European Union countries could help identify best practices in policy language and accessibility. One potential solution at a national level would be to implement a standardized, cross-provincial performance framework for disability care services and facilities in Austria, as recommended by the Austrian Disability Council [44], to make them more comparable with international concepts and structures. Having nationwide guidelines for facilities for people with disabilities would not only facilitate international comparisons, but it would also enable measures to promote PA to be implemented uniformly and in a holistic way. Bodde and Seo [45] emphasize in their review of social and environmental barriers to PA for adults with intellectual disabilities the development of guidelines in institutions for people with intellectual disabilities as a key approach to removing financial, structural, transportation-related, and education-related barriers.

The document analysis also considered three of Austria’s ten health goals, which provide the basis for a comprehensive health promotion policy until 2032. The analysis covered Health Goal 1 (‘Creating health-promoting living and working conditions together’) [32], Health Goal 3 (‘Strengthening the health literacy of the population’) [28] and Health Goal 8 (‘Fostering healthy and safe movement in daily life’) [23]. Significant variation was observed in the number of coded segments within the “Austrian health goals” document group: Health Goal 1 [32] was represented by only four segments, Health Goal 3 [28] by no coded segments, and Health Goal 8 [23] by 184. These differences can be explained by the respective health goals’ content focus. Health Goal 8 [23] directly addresses the health-promoting effects of PA in everyday life and is therefore much more relevant in the context of the examined documents.

As previously mentioned, the documents in the “Austrian documents on PA and disabilities” group contain numerous references to terms and objectives relating to the promotion of PA based on predefined criteria. Of note, here are the two central documents on the promotion of PA in Austria: Austrian recommendations for health promotion through PA, which contains a total of 501 coded segments, and the national action plan for PA, which contains 394 coded segments. By contrast, Austrian documents from the same group whose content focuses primarily on people with disabilities refer much less frequently to the promotion of PA. For instance, the analysis also included a national action plan for the implementation of the UNCRPD [35]. However, only 13 references to measures or objectives for promoting PA were identified in this document.

The results of the analysis also demonstrate that the five WHO documents from the document group “WHO guidelines and documents” examined contain numerous references to terminology and objectives related to the promotion of PA, and therefore represent a solid starting point for encouraging health-enhancing PA. This also applies to the target group of people with intellectual disabilities specifically, for example, as the document “Guidelines on Physical Activity and Sedentary Behaviour” [15] explicitly includes this target group. To specifically improve documents with few references to PA promotion, policymakers and society can draw on international guidelines as well as WHO strategy and action concepts. Especially in the service descriptions of disability assistance services and facilities, adaptations could provide added value for promoting PA among people with intellectual disabilities, as many of them use disability assistance services. Knowledge about health-promoting PA and its positive physical and psychosocial effects can thus be disseminated and made accessible to more people with intellectual disabilities.

As part of the document analysis, it was also examined whether the documents were available in easy-to-read language. Apart from the German version of the UNCRPD [30], it was hard to find other accessible documents in easy-to-understand language that corresponded to the original in terms of content and had similar bibliographic characteristics without extensive online research. This may represents a barrier for people with intellectual disabilities [46,47]. A national mandate for publishing health guidelines in plain language could significantly improve access. This would strengthen the significant role of public health recommendations as important guidelines for promoting PA. In two publications [39,40], the key recommendations are also provided in easy-to-read language, making them more accessible to people with intellectual disabilities. As Walther [47] emphasizes, independent access to health-related information is essential for the self-efficacy of this target group—also in the context of PA promotion. In addition to providing knowledge on topics related to PA in easy-to-read language [47], the literature increasingly emphasizes the importance of structural concepts for promoting PA [45] as well as the central role of supporters and caregivers is emphasized in the case of promoting PA for people with intellectual disabilities [5,45,48]. An increase in PA is particularly successful when communication is improved and attitudes toward exercise are positively influenced. Additionally, PA is understood as an integral part of everyday life [48]. People in the environment of individuals with intellectual disabilities, including employees in residential facilities, service providers, and political decision-makers, can make a significant contribution to breaking down barriers. This enables individuals with intellectual disabilities to make independent decisions about their health, consistent with the concept of empowerment [45].

## 5. Limitations

When conducting the document analysis, a systematic approach based on a specific category system in combination with a correspondence table was chosen to examine the documents specifically for the topic of PA promotion. An impact objective of Austria’s eighth health goal served as the content basis, on which the documents in the sample were analyzed for relevant terms and objectives.

One limitation of the study is that no automated procedure was used for the frequency analysis or the analysis of the frequency of references. Although a fully automated evaluation was deliberately avoided to enable a more context-dependent content analysis, a standardized automated procedure might have led to deviating results.

A further limitation lies in the selection of documents. The focus was predominantly on Austrian documents, while international political and institutional guidelines and service descriptions of disability care services and facilities, except for the UNCRPD, were not included in the sample. Further analysis with a stronger focus on international documents could provide valuable insights into how PA promotion is implemented within disability care in other countries.

## 6. Conclusions

The document analysis related to PA promotion for people with (intellectual) disabilities shows that the documents examined in the sample (N = 19) contain coded segments with varying frequency. This indicates that the objectives and concepts of PA promotion are represented to varying degrees in the respective documents. It is striking that in central Austrian political and institutional guidelines, laws and handouts on the inclusive participation of people with (intellectual) disabilities—as well as in selected service descriptions of facilities for people with disabilities—few to no references to the promotion of PA and corresponding terminology and objectives were made.

The promotion of PA is most clearly addressed in international WHO guidelines and directives as well as in national strategy and action plans for the promotion of PA in Austria. Some of these documents also explicitly mention people with (intellectual) disabilities as a target group.

It is particularly important for people with intellectual disabilities that both international and national documents on PA promotion are available in easy-to-understand language. This would not only be important in terms of an inclusive approach but could also strengthen the self-efficacy of this group of people by giving them independent access to knowledge and information on the topic of PA.

## Figures and Tables

**Table 1:** Correspondence table of the document analysis *.

General rules	Segments that appear in the title page, imprint, table of contents and bibliography, in acknowledgements, headers and footers and in appendices (LEVO StBHG [25] is excluded here due to the structure of the document) are not coded. Segments in illustrations and figures are not coded (headings are coded if they are outside the illustrations and figures). Content in tables is coded if the coded elements can be meaningfully coded. Titles within the body text are coded; names of organizations, associations, ministries, etc., are not coded. If terms and segments can be assigned to more than one coding, only one coding is generally used, namely the coding that is considered to have a higher weighting (e.g., recommended levels of physical activity is assigned to the subcategory *Recommendation on PA* and not *(regular) PA.* In case of any discrepancies in assigning the codes, a consensual process was used by the authors. Terms in the plural are coded without changes; terms in the genitive are smoothed. The size of the coded elements is kept small for the main categories of *Promoting PA*, *Forms and types of PA* and *PA settings* (the number of words per segment was between one and twelve words). The segments of the subcategories *Knowledge* are coded more extensively, so that a coded segment can encompass an entire paragraph. A coding can therefore contain several pieces of information. However, empty lines within segments are not coded. New coding is carried out for a new paragraph (multiple coding possible).	
**Subcategory/Coded Segments**	**Coding Rule**	No Coding **
*Promoting PA*		
*Promoting PA*	The term is coded if the promotion of physical activity is addressed. (e.g., promotion of PA, culture that promotes PA, promotion of regular PA)	No coding if measures for the promotion of coping with life are described (e.g., promotion of mobility), forms of promotion without explicit reference to the promotion of PA (e.g., promotion of freedom of action) Workplace health promotions were also not coded.
*Knowledge*		
*General/non-group-specific knowledge*	Coding if text element conveys knowledge about positive effects of PA and why PA is important; knowledge is general and not group-specific (following subcategories)	Knowledge about economic benefits and costs is not coded
*Group-specific knowledge*	Coded if the positive health effects of PA are described in the text and explicit reference is made to one of these target groups: *PA for people with disabilities,* *Children & adolescents, Adults, Older adults, Adults & older adults with chronic conditions,* *Pregnant & postpartum women*	Knowledge about economic benefits and costs is not coded
*Forms and types of PA*		
*Levels of PA*	Coded segments in addition to PA levels are, e.g., PA levels, levels of PA and physical activity levels	Exercise workload, exercise units
*Amounts of PA*	Coded segments in addition to the amount of PA are, for example, the amount of PA, or the recommended amount of PA	
*Movement behavior*	Coded segments besides movement behavior are, e.g., activity behavior	Not coded if the behavior is different
*(regular) PA*	Also coded for PA, sporting activity, recreational sports activities, physically active exercise	e.g., PA behavior, recreational sporting activity, physical training, regular strength training, active movement, physically so active
*Recommendation on PA*	Also coded for health-effective PA recommendations; recommendation alone is also coded if PA recommendations are meant	e.g., public health recommendation, recommended
*PA settings*		
*Movement options & offers*	PA opportunities and PA offers can also be coded separately; also coded for recreational facilities, offers around popular and school sports	
*Leisure & sporting activities*	Leisure activities and sports activities can also be coded separately; also coded for leisure activities, individual sports activities	e.g., endurance activities during leisure time, physically active leisure time, exercise during leisure time
*Physical education*	Physical education and exercise classes can also be coded separately.	
*Sports clubs*	Deviating terms are not coded	Sports umbrella organizations
*Space for movement*	Also coded for rooms for safe movement, movement and meeting rooms	
*Sports facilities*	Also coded for sports, recreation and tourism facilities	

Note: * The correspondence table was translated into English; therefore, the German terms were not considered. ** Supplementary for some subcategories.

**Table 2:** Distribution of documents and frequencies of coded segments according to main categories.

Documents *	Total	*Promoting PA*	N **	*Knowledge*	N	*Forms and Types of PA*	N	*PA Settings*	N
UNCRPD (German translation) (2016) [30]	6	0	-	0	-	1	1	1	5
LEVO (2015, version 2025) [25]	7	1	2	0	-	1	2	1	3
Tyrolean disabled benefits catalogue 2015) [31]	16	1	1	1	1	1	6	1	8
Health goals 1 (2017) [32]	4	1	2	0	-	0	-	1	2
Health goals 8 (2022) [23]	184	1	44	1	30	1	43	1	67
Austrian recommendations on PA (2020)[24]	501	1	30	1	101	1	332	1	38
NAP.b (2024) [33]	394	1	113	1	38	1	124	1	113
NAP 2022–2023 implementation report (2024) [34]	13	1	1	0	-	1	2	1	10
NAP disability 2022–2030 (2022) [35]	17	0		1	1	1	2	1	14
Motion monitoring 2022 (2022) [36]	139	1	7	1	3	1	110	1	19
GPASB (2020) [15]	220	1	1	1	34	1	185	0	-
GAPPA (2018) [37]	149	1	9	1	19	1	117	1	4
PA report (2022) [20]	559	1	43	1	18	1	493	1	5
GSDPAH (2004) [21]	123	1	4	1	10	1	109	0	-
Incl. Sports Federation (2023) [38]	304	1	5	1	3	1	34	1	262
**Total**	2636	13	262	11	258	14	1560	13	550

Note: * The four documents in which no references for promoting PA could be identified were not included in the table. ** Number of references.

**Table 3:** Examples of coded segments of the subcategory *PA promotion*.

Document	Segments
Global Status, Report on Physical Activity ([20], p. 74)	Promoting and enabling physical activity
Global Action Plan on Physical Activity ([37], p. 37)	physical activity programmes and promotion
Global Strategy on Diet, Physical Activity and Health ([21], p. 9)	promoting physical activity

**Table 4:** Examples of coded segments of the subcategory *PA for people with disabilities*.

Document	Segments
Global Status, Report on Physical Activity ([20], p. 77)	People living with disability are less likely to be physically active compared to those without disability […]. This increases their NCD risk, while also being potentially detrimental for their mental health and social well-being […]. Providing opportunities for inclusion in physical activity for people living with disability can help eliminate such barriers by changing perceptions, emphasizing strengths and abilities, promoting personal resilience, and having a longer-term impact on inclusion in society
Guidelines in Physical Activity and Sedentary Behaviour ([15], p. 12)	Additional benefits of physical activity to health outcomes for those living with disability include improved cognition in individuals with diseases or disorders that impair cognitive function, including attention-deficit/hyperactivity disorder (ADHD); improvements in physical function may occur in children with intellectual disability

## Data Availability

Data is availability upon reasonable request by the correspondent author.

## Abbreviations

The following abbreviations are used in this manuscript:

GAPPA	Global Action Plan on Physical Activity
GPASB	Guidelines on Physical Activity and Sedentary Behaviour
GSDPAH	Global Strategy on Diet, Physical Activity and Health
Incl. Sports Federation	Inclusive, sustainable, welcoming national sports federations. Health-promoting sports federation implementation guidance
LEVO	Service and Remuneration Regulation [Leistungs- und Entgeltverordnung]
NAP	National Action Plan
NAP.b	National Action Plan for Physical Activity
PA	Physical activity
PA report	Global Status Report on Physical Activity 2022
UNCRPD	United Nations Convention on the Rights of Persons with Disability
WHO	World Health Organization
